# Supplementary material for: Seroprevalence and Risk Factors Associated with Leishmania Infection in Dogs from Portugal
Source: Microorganisms. 2022 Nov 15;10(11):2262. doi: 10.3390/microorganisms10112262 (PMC9695918; doi:10.3390/microorganisms10112262)
Supplement: Supplementary file 1 [file microorganisms-10-02262-s001.zip › microorganisms-2001058-supplementary.pdf]

Supplementary Materials

Seroprevalence and Risk Factors Associated with *Leishmania* Infection in Dogs from Portugal

Seroprevalence and Risk Factors Associated with *Leishmania* Infection in Dogs from Portugal

**Maria Almeida [1], Carla Maia [1,2], José M. Cristóvão [1], Cátia Morgado [3], Inês Barbosa [4], Ruben Foj Ibars [3],
Lenea Campino [1], Luzia Gonçalves [1,5,6] and Sofia Cortes [1,2,*]**

1 Instituto de Higiene e Medicina Tropical (IHMT), Universidade Nova de Lisboa (UNL), Rua da Junqueira, 100, 1349-008 Lisboa, Portugal
2 Global Health and Tropical Medicine (GHTM), Instituto de Higiene e Medicina Tropical (IHMT), Universidade Nova de Lisboa (UNL), Rua da Junqueira, 100, 1349-008 Lisboa, Portugal
3 LETI Pharma S.L.U., 08038 Barcelona, Spain
4 MSD Animal Health Lda, 2770-192 Paço de Arcos, Portugal
5 Centro de Estatística e Aplicações da Universidade de Lisboa (CEAUL), Faculdade de Ciências, Universidade de Lisboa, 1749-016 Lisboa, Portugal
6 z-Stat4life, Cowork do Palácio Baldaya, 1549-0111349-008 Lisboa, Portugal
* Correspondence: scortes@ihmt.unl.pt

Table S1. Veterinary clinics ("Centros de atendimento medico-veterinário") enrolled in the national survey.

Region	District	Veterinary clinics
North	Viana do Castelo	Clínica Médico-Veterinária d'Areosa
		Clínica Veterinária de Viana
		Clivetviana - Praia de Âncora
	Vila Real	Clinica Veterinária de Chaves
		Clínica Veterinária de Valpaços
		Clinica Veterinária Marãovet
		Consultório Veterinário Dra Marta Rebelo
		Reguavet Clínica Veterinária
	Bragança	Clínica Veterinária Dr. Duarte Diz Lopes
		Clínica Veterinária Terra Quente, Lda
		Consultório Veterinário Animal SOS
		Consultório Veterinário Dr.ª Isabel Lameira
		Dr4Patas
	Braga	Centro Veterinário de Merelim
		Clínica Veterinária de Areia
		Clínica Veterinária de Martim

		Clínica Veterinária Saúde Animal<br>Vetbasto Serviços Médico-Veterinários, Lda<br>Centro Veterinário do Parque
	Porto	Centro Veterinário de Amarante<br>Vallis Vet- Centro Veterinário<br>Clínica Veterinária da Areosa<br>LEÇAVET- Clínica Veterinária<br>Consultório Veterinário do Freixieiro<br>Clínica Veterinária de Oldrões<br>SOS Patinhas- Clinica medico veterinária Centralparl, Lda
Centre	Aveiro	Centro Veterinário de Oliveira do Bairro<br>Clínica Veterinária do Vouga<br>O MEU VET - Clínica Veterinária<br>Clínica Veterinária da Vagueira<br>Termasvet - Consultório Veterinário
	Viseu	Caniféli - Clínica veterinária Lda - Tondela<br>Caniféli - Clínica veterinária Lda - Santa Comba Dão<br>Clínica Veterinária Douro Sul Lda-Lamego<br>Clínica Veterinária Douro Sul Lda-Peso da Régua<br>Clínica Veterinária Douro Sul Lda-Tarouca<br>Rosela Clínica Veterinária
	Guarda	Animalvet Hospital Veterinário<br>Clínica Veterinária Serra da Estrela
	Coimbra	Centro de Saúde Animal<br>Centro Veterinário Cantanhede- Dr. André Caldeira<br>Centro Veterinário Vale das Flores, Lda<br>Clínica veterinária das Nogueiras<br>Oficina dos Animais - Clínica Veterinária Lda<br>VetConímbriga, Lda<br>Vetsoure - Clínica Veterinária, Lda<br>VetSunpi
	Castelo Branco	Clínica Veterinária de Castelo Branco (Hupera)<br>Fundão Vet - Centro Veterinário<br>Clínica Veterinária Vetbeirão
	Leiria	Centro Veterinário de São Jorge<br>Clínica Veterinária do Lis<br>Clínica Veterinária Milagres<br>Clínica Veterinária São Romão<br>Farmanimal Centro Médico Veterinário<br>Vetfigueiró<br>Clínica Veterinária Pombalvet
Lisbon and Tagus Valley	Lisboa	Animais & Cãopanhia Veterinários<br>Animalcare - Centro Veterinário Póvoa da Galega<br>Clínica Veterinária do Lambert<br>Clínica Veterinária Mascotes Sortudas

		Consultório Veterinário da Galiza - Maria Gonçalves<br>Hospital Veterinário Vasco da Gama-Parque das Nações<br>Hospital Veterinário Vasco da Gama- Odivelas<br>Hospital Veterinário Vasco da Gama - Forte da Casa<br>Manuel Dargent Figueiredo<br>Pet´Spot/MVF-Veterinário de Família Lda<br>Vetzoo de Famões<br>Vip Pets Arruda dos Vinhos
	Santarém	Animabilis<br>Bicos, Pêlos e Patas<br>Clínica Veterinária Cão d'Amor<br>Centro Veterinário de Alcanede<br>Clínica Veterinária de Ourém<br>Clinica Veterinaria Torres Pet<br>Hospital Veterinário Tutivete - Centro Cirúrgico de Santarém
	Setúbal	Centro Veterinário da Costa Vicentina<br>Doutoras dos Animais<br>Vet Santa Maria<br>VETSET - Hospital Veterinário<br>Vetzone - Consultório Veterinário de Grândola<br>Vetzone - Consultório Veterinário de Santiago do Cacém
Alentejo	Portalegre	Clínica Veterinária das Laranjeiras<br>Clínica Veterinária de Santo Onofre
	Évora	Clínica Veterinária 112 Animal<br>Hospital Veterinário Muralha de Évora<br>Optivet<br>Vetviana, Consultório Veterinário, Lda<br>Vetvila - Clínica Veterinária de Vila Viçosa
	Beja	Consultório Veterinário Dr. Nuno Costa Neves<br>Refúgio Animal<br>SingaVet- Centro Veterinário<br>Vetmoura - Centro Veterinário De Moura, Lda
Algarve	Faro	Algarvet - Centro Veterinário dos Olhos de Água<br>Clínica Veterinária de Loulé<br>Villapet<br>Clínica Veterinária da Guia<br>Império do Animal

Table S2. Minimum stratified proportional sample sizes by geographical region (NUTS2) and District.

Region	District	Dog's population per Region (A)	Dog's population per District[1] (B)	Minimum Region sample size[2] (C)	Districts stratified proportional sampling	
					Ratio (D=B/A)	Samples (C x D)
**North**		669 462		177		
	Viana do Castelo		65 512		0.10	17
	Vila Real		51 062		0.08	14
	Bragança		47 778		0.07	13
	Braga		187 709		0.28	50
	Porto		317 401		0.47	83
**Centre**		590 478		360		
	Aveiro		147 353		0.25	85
	Viseu		94 621		0.16	54
	Guarda		44 576		0.08	26
	Coimbra		113 316		0.19	65
	Castelo Branco		44 331		0.08	26
	Leiria		146 281		0.25	84
**Lisbon and Tagus Valley**		788 090		261		
	Lisboa		464 115		0.59	154
	Santarém		134 678		0.17	44
	Setúbal		189 297		0.24	63
**Alentejo**		158 270		417		
	Portalegre		43 016		0.27	113
	Beja		54 619		0.35	144
	Évora		60 632		0.38	160
**Algarve**		160 671		219		
	Faro		160 671		1	219
**Total**			2 366 968	1414		1414

[1] SIAC 2020 data; [2]Minimum stratified proportional sampling per Region calculated on Epitools (https://epitools.ausvet.com.au/), considering: 95% CI, 3% precision; DAT 93% sensitivity and 100% specificity according to Ferreira et al [20] and Regions true prevalence of according to Cortes et al [13]. NUTS, Nomenclature of Territorial Units for Statistics.

Table S3. Geographical distribution of vaccinated animals per vaccine.

Region	District	Total*		Dogs Vaccinated with known vaccine		
		No. Dogs Tested	No. Vaccinated Dogs (%)	Total	Letifend® (%)	Canileish® (%)
**North**		452	23 (5.1%)	13	11 (84.6%)	2 (15.4%)
	Viana do Castelo	51	0 (0.0%)	0	0 (0.0%)	0 (0.0%)
	Vila Real	104	3 (2.9%)	3	2 (66.7%)	1 (33.3%)
	Bragança	80	6 (7.5%)	1	1 (100.0%)	0 (0.0%)
	Braga	91	3 (3.2%)	2	2 (100.0%)	0 (0.0%)
	Porto	126	11 (8.7%)	7	6 (85.7%)	1 (14.3%)
**Centre**		512	64 (12.5%)	56	38 (67.9%)	18 (32.1%)
	Aveiro	87	5 (5.7%)	4	4 (100.0%)	0 (0.0%)
	Viseu	82	6 (7.3%)	6	6 (100.0%)	0 (0.0%)
	Guarda	39	8 (20.5%)	7	5 (71.4%)	2 (28.6%)
	Coimbra	129	19 (14.7%)	16	7 (43.8%)	9 (56.2%)
	Castelo Branco	70	16 (22.9%)	13	10 (76.9%)	3 (23.1%)
	Leiria	105	10 (9.5%)	10	6 (60.0%)	4 (40.0%)
**Lisbon and Tagus Valley**		490	115 (23.5%)	90	66 (73.3%)	24 (26.7%)
	Lisboa	205	53 (25.9%)	51	35 (68.6%)	16 (31.4%)
	Santarém	161	28 (17.4%)	11	9 (81.8%)	2 (18.2%)
	Setúbal	124	34 (27.4%)	28	22 (78.6%)	6 (21.4%)
**Alentejo**		236	26 (11.0%)	23	11 (47.8%)	12 (52.2%)
	Portalegre	60	8 (13.3%)	5	0 (0.0%)	5 (100.0%)
	Beja	59	4 (6.8%)	4	3 (75.0%)	1 (25.0%)
	Évora	117	14 (12.0%)	14	8 (57.1%)	6 (42.9%)
**Algarve**		134	43 (32.1%)	23	16 (69.6%)	7 (30.4%)
	Faro	134	43 (32.1%)	23	16 (69.6%)	7 (30.4%)
**Total**		1824	271 (14.9%)	205	142 (69.3%)	63 (30.7%)

*includes vaccinated dogs where the vaccine is unknown.

Table S4. Results of DAT titers for positive samples (n=1860).

DAT titre	No. positive dogs	% of seropositive dogs
400	38	17.5
800	28	12.9
1600	5	2.3
3200	7	3.2
6400	4	1.8
12800	1	0.5
25600	134	61.8
Total	217	11.7

Table S5. True seroprevalence for *Leishmania* infection per geographic region and District excluding dogs vaccinated with Canileish®.

Geographic Region/District	No. tested dogs	No. seropositives (%)	% True seroprevalence	95% CI
**North**	456	41 (9.0)	9.5	7.0 – 12.6
Viana do Castelo[1]	51	0 (0.0)	0.0	0.0 – 0.1
Braga[1]	93	6 (6.5)	6.8	3.2 – 14.1
Vila Real[2]	103	12 (11.7)	12.3	7.2 – 20.3
Bragança[2]	82	12 (14.6)	15.4	9.0 – 25.1
Porto[1]	127	11 (8.7)	9.1	5.2 – 15.6
**Centre**	511	50 (9.8)	10.3	7.9 – 13.3
Aveiro[1]	91	1 (1.1)	1.2	0.0 – 6.3
Viseu[2]	86	11 (12.8)	13.5	7.7 – 22.6
Guarda[2]	37	6 (16.2)	17.1	8.1 – 32.8
Coimbra[1]	122	13 (10.7)	11.2	6.7 – 18.3
Castelo Branco[2]	69	17 (24.6)	25.9	16.8 – 37.8
Leiria[2]	106	2 (1.9)	2.0	0.5 – 7.0
**Lisbon and Tagus Valley**	472	44 (9.3)	9.8	7.4 – 12.9
Lisboa[1]	194	13 (6.7)	7.0	4.1 – 11.7
Santarém[2]	159	15 (9.4)	9.9	9.1 – 15.8
Setúbal[1]	119	16 (13.4)	14.2	8.9 – 21.8
**Alentejo**	230	33 (14.3)	15.1	11.0 – 20.5
	55	15 (27.3)	28.7	18.2 – 42.4
Portalegre[2]	59	8 (13.6)	14.3	7.4 – 25.8
Beja[2]	116	10 (8.6)	9.1	5.0 – 15.9

Évora[2]				
**Algarve**	128	19 (14.8)	15.6	10.2 – 23.2
Faro[1]	128	19 (14.8)	15.6	10.2 – 23.2
**Total**	1797	187 (10.4)	11.0	9.6 – 12.5

[1] Littoral District; [2] Interior District; CI, confidence interval.

Table S6. Most frequent dog breeds[1].

**Breed**[1]	**No. dogs**
Labrador Retriever	175
German Sheperd	77
French Bulldog	65
Pinscher	59
Podengo[2]	46
Beagle	40
Golden Retriever	33
Rafeiro Alentejano[2]	27
Epagnuel Breton	26
Chihuahua	24
Serra da Estrela[2]	23

[1]Other breeds (n= 68) and mixed breeds had each less than 22 dogs; [2]autochthonous breeds.

Table S7. Most frequent reported clinical signs compatible with canine leishmaniosis.

**Clinical Sign**	**No. dogs**
Loss of appetite / weight loss	13
Ulcers, wounds, and other unspecified skin lesions	11
Alopecia	7
Onychogryphosis	6
Hepatic Alterations	6
Locomotion difficulties/Articular pain	4
Ocular manifestations	3
Pruritus	1
Dermatitis	1
Lymphadenomegaly	1

A.

Consentimento informado de participação no projeto "Semana da Leishmaniose Canina"

A leishmaniose canina é uma doença causada por um parasita (*Leishmania*) transmitido por um inseto, o flebótomo, que pode causar, no animal, perda de apetite, perda de peso, feridas na pele, conjuntivite e, até mesmo, a morte.

O que pretendemos nós fazer?

Fazer um rastreio para avaliar cães expostos ao parasita responsável pela leishmaniose canina em Portugal.

O que tenho de fazer para o meu cão participar no estudo?

Dar o meu consentimento para que, uma pequena amostra de sangue do meu cão, que poderá ser colhida por outras razões (ex.: análises de rotina, despiste de outra doença, avaliação pré-cirúrgica), seja alvo de uma análise prevista neste estudo.

Quais são os benefícios do estudo?

O estudo permitirá detetar cães expostos ao parasita em todo o país. No caso de o resultado ser positivo, este será comunicado ao médico veterinário no espaço de dois meses. Deste modo, o clínico pode avaliar o resultado e, caso ache necessário, sugerir procedimentos adicionais para o diagnóstico definitivo.

Consentimento informado

Ao aceitar que o meu cão participe neste estudo, declaro que me foram comunicadas, pelo médico veterinário assistente, informações suficientes relativas ao mesmo. A participação do(s) meu(s) cão (cães) de que sou detentor garante-me, ainda, o direito de colocar as perguntas que achar convenientes e de obter informação adicional relacionada com o estudo em questão. A análise para este rastreio é-me disponibilizada gratuitamente.

Nome do canídeo:____

Assinatura do detentor do animal:_________________________________

Local e Data: ______________________, ____ de _________________ de 2021

Eu ____ declaro ter efetuado a colheita de sangue ao canídeo acima identificado e ter explicado ao seu detentor o âmbito e os passos do estudo Semana da Leishmaniose Canina.

B.

Semana da Leishmaniose Canina

Dados do canídeo

CAMV:	Médico Veterinário:
Data colheita (dd/mm/aa):	Amostra nº (ref. papel filtro):
Nome do Canídeo:	Sexo: M ◯ / F ◯
Data nasc. (ou idade aprox.):	Raça:
Pelagem: curta ◯ / média ◯ / comprida ◯	
Localidade onde vive o animal:	
Freguesia onde vive o animal:	
Concelho onde vive o animal:	
O proprietário teve conhecimento do rastreio gratuito: antes de chegar ao CAMV ◯ / depois de chegar ao CAMV ◯	
O animal permanece: exclusivamente dentro de casa ◯ / a maior parte do tempo dentro de casa ◯ / igualmente dentro e fora de casa ◯ / a maior parte do tempo fora de casa ◯ / exclusivamente fora de casa ◯	
O animal é tratado com insecticidas/repelentes ? Não ◯ / Sim ◯ Qual (Quais)? Periodicidade: Mensal ◯ 3 em 3 meses ◯ 8 em 8 meses ◯ anual ◯	
O animal é vacinado? Não ◯ Sim ◯ CaniLeish® ◯ Letifend® ◯ Data da última dose:	
Aspectos clínicos: Animal assintomático ◯ / Clinicamente suspeito de Leishmaniose canina ◯	
O animal tomou medicação nos últimos 15 dias? Não ◯ Sim ◯ Se sim, qual?	
Sinais físicos e laboratoriais eventualmente presentes (discriminar sff.):	
Referência do Proprietário:	

Critérios de exclusão: cães com idade inferior a 6 meses; cães vacinados há menos de 6 meses com CaniLeish.

Figure S1. Informed consent (A) and a questionnaire (B) for the collection of dog's blood and epidemiological variables (in Portuguese).
